# The value of maternity care in Queensland, 2012–18, based on an analysis of administrative data: a retrospective observational study

**DOI:** 10.5694/mja2.52156

**Published:** 2023-11-08

**Authors:** Emily J Callander, Joanne C Enticott, Bonnie Eklom, Jenny Gamble, Helena J Teede

**Affiliations:** ^1^ University of Technology Sydney Sydney NSW; ^2^ Monash Centre for Health Research and Implementation Monash University Melbourne VIC; ^3^ Coventry University Coventry the United Kingdom

**Keywords:** Maternal health, Perinatal, Pregnancy, Health services research, Health financing, Financial management, Economics, medical

## Abstract

**Objective:**

To quantify the value of maternity health care — the relationship of outcomes to costs — in Queensland during 2012–18.

**Study design:**

Retrospective observational study; analysis of Queensland Perinatal Data Collection data linked with the Queensland Health Admitted Patient, Non‐Admitted Patient, and Emergency Data Collections, and with the Medicare Benefits Schedule (MBS) and Pharmaceutical Benefits Scheme (PBS) databases.

**Setting, participants:**

All births in Queensland during 1 July 2012 – 30 June 2018.

**Main outcome measures:**

Maternity care costs per birth (reported in 2021–22 Australian dollars), both overall and by funder type (public hospital funders, MBS, PBS, private health insurers, out‐of‐pocket costs); value of care, defined as total cost per positive birth outcome (composite measure).

**Results:**

The mean cost per birth (all funders) increased from $20 471 (standard deviation [SD], $17 513) during the second half of 2012 to $30 000 (SD, $22 323) during the first half of 2018; the annual total costs for all births increased from $1.31 billion to $1.84 billion, despite a slight decline in the total number of births. In a mixed effects linear analysis adjusted for demographic, clinical, and birth characteristics, the mean total cost per birth in the second half of 2018 was $9493 higher (99.9% confidence interval, $8930–10 056) than during the first half of 2012. The proportion of births that did not satisfy our criteria for a positive birth outcome increased from 27.1% (8404 births) during the second half of 2012 to 30.5% (9041 births) during the first half of 2018.

**Conclusion:**

The costs of maternity care have increased in Queensland, and many adverse birth outcomes have become more frequent. Broad clinical collaboration, effective prevention and treatment strategies, as well as maternal health services focused on all dimensions of value, are needed to ensure the quality and viability of maternity care in Australia.

**Summary box**

**The known**: Medical intervention rates in maternity care, a high volume, high cost area of health care, need to be reduced to contain costs.

**The new**: During 2012–18, the demographic and clinical characteristics of women giving birth in Queensland changed markedly, and the proportion of births without any adverse outcomes declined from 72.9% to 69.5%. Further, the costs of maternity care increased at a rate that cannot be sustained indefinitely.

**The implications**: Delivering high value, high quality maternity care requires strategies for containing costs while maintaining its quality.

Value‐based care is not just a question of cost. It also takes the experience of both the patient and the care provider into account, as well as the quality of care and its outcomes, efficiency, and sustainability.^1^ Value‐based care is more focused on community need than on health care providers, services, and systems.^2^ In high income countries, it has been estimated that 60% of care is evidence‐based, 30% is of low value, and 10% is harmful.^3^ The problem of low value care is particularly pressing in maternity care, a high volume, high cost area of health care in which intervention rates are rising.^4^

Quality is a key component of high value care. Challenges to providing quality maternity care include the changing demographic characteristics of women giving birth, including increasing age and greater frequency of obesity and weight‐related conditions,^5^, ^6^ each associated with greater risk of adverse outcomes.^7^, ^8^ All women are entitled to high value maternity care, and optimal outcomes and value must be achieved in the face of increasing clinical complexity.

Unprecedented government health care spending during the coronavirus disease 2019 (COVID‐19) pandemic, leading to record debt and inflation levels in many countries, has inevitably been followed by the rationalising of expenditure.^9^ As spending on health care changes, value‐based care is even more critical for maintaining quality and equity. How better value could be achieved in maternity care has been discussed for some time,^10^, ^11^ but there have been few assessments of its current value or changes over time that would assist health services monitor their performance, compare it with that of similar services, or develop strategies for improving high value care in priority areas.

The quality of the publicly funded health care system in Australia, which is similar to those of many high income countries, is among the highest in the world.^12^ However, not all women have equal access to maternity care.^13^ Further, intervention rates during pregnancy are increasing; it is projected, for example, that 45% of births in Australia and New Zealand in 2030 will be by caesarean delivery.^14^ We therefore analysed administrative data routinely collected for monitoring the value of maternity care in Australia, quantifying changes over time in perinatal outcomes for women and their infants, and in maternity care costs. Our analysis is based on the definition of value proposed by Porter,^2^ which defines value as the outcomes achieved at a given level of expenditure.^15^

## Methods

For our retrospective observational study, we analysed data from a population dataset that comprised linked routine administrative data for all births in Queensland during 1 July 2012 – 30 June 2018.^16^ Women who gave birth and their infants were identified in the Queensland Perinatal Data Collection, which records the demographic and clinical characteristics of women before and during pregnancy, as well as information about the birth and postpartum events. These data were linked with data for both women and their infants, from the start of pregnancy until 30 June 2019, in the Queensland Health Admitted Patient Data Collection (which records all inpatient events in private and public hospitals), the Queensland Health Non‐Admitted Patient Data Collection (all outpatient services) and the Emergency Data Collection (all emergency department presentations). The data were then linked with Medicare Benefits Schedule (MBS) and Pharmaceutical Benefits Scheme (PBS) claims records to encompass all community‐based services (including general practitioner and specialist consultations, pathology services, and diagnostic tests and imaging), private hospital services, and prescription pharmaceutical dispensing (further details: Supporting Information). De‐identified data were supplied to the authors by the Australian Institute of Health and Welfare and the Queensland Health Statistical Services Branch.

### Health care costs

We included costs for all health services for women during their pregnancy and until their discharge from hospital after giving birth, and for infants from birth to their discharge from hospital, overall and by health funder type (public hospital funders, MBS, PBS, private health insurers, and out‐of‐pocket costs). Analyses based on mean costs are reported per birth; that is, the births of multiple babies from a single pregnancy were considered as separate births. Analyses based on summed costs are reported per pregnancy to avoid double counting of multiple births. All costs were adjusted for inflation and are reported in 2021–22 Australian dollars using the Reserve Bank of Australia inflation calculator (https://www.rba.gov.au/calculator/financialYearDecimal.html).

### Health care outcomes

We developed a composite birth outcome measure in an earlier study,^17^ after two rounds of community consultation, based on data routinely collected in accordance with the values and principles outlined in the *Australian woman‐centred care: strategic directions for Australian maternity services strategy*.^18^ Specifically, a positive birth outcome was defined as one for which none of the following adverse outcomes were recorded in the linked dataset: stillbirth; neonatal death (within thirty days of birth); admission to a special care nursery or neonatal intensive care unit; Apgar score at five minutes below 7; hypoxic–ischaemic encephalopathy; infant birth trauma (brachial plexus injury, fractured clavicle or humerus); intrauterine hypoxia; other perinatal conditions (meconium aspiration syndrome, congenital pneumonia or respiratory distress syndrome); third or fourth degree perineal tear; intra‐ or postpartum haemorrhage; retained placenta; rupture of the uterus.

### Statistical analysis

We summarise the demographic and clinical characteristics of women who gave birth, perinatal outcomes, and cost per birth as descriptive statistics by year. We report the proportions of women with positive birth outcomes by year. The statistical distribution of cost per birth is reported for all births by year, as is the mean cost by funder for each year. The cost (for all births) per positive birth outcome was calculated. Changes in demographic and clinical characteristics, outcomes, and costs are reported as differences relative to 2012–13, and the statistical significance of changes was assessed in Cochrane–Armitage, Cochran–Mantel–Haenszel, or Jonckheere–Terpstra tests for trend.

We examined change over time in total costs per birth in a mixed effects linear model, with a repeated measure for mothers and a year term, and adjusted for mother's age, body mass index, Indigenous status, country of birth (Australia, other), parity, pregnancy type (singleton, multiple), pre‐existing or gestational diabetes, hypertension, pre‐eclampsia, iatrogenic birth at 34–37 weeks’ gestation, spontaneous birth at 34–37 weeks’ gestation, iatrogenic birth beyond 37 weeks, and congenital defects at birth. All variance inflation factor values were below 10 and all tolerance values above 0.1, indicating that collinearity between the included variables was not statistically significant. All analyses were conducted in SAS 9.4; *P* < 0.001 was deemed statistically significant.

### Ethics approval

Our study was conducted in accordance with the Declaration of Helsinki. The human research ethics committees of the Townsville Hospital and Health Service (HREC/16/QTHS/223) and the Australian Institute of Health and Welfare (EO2017‐1‐338) approved access to and linkage and analysis of the raw data for which they are the custodians.

## Results

The Queensland Perinatal Data Collection recorded 365 053 births during 1 July 2012 – 30 June 2018. The proportion of mothers under 20 years of age when they gave birth declined from 4.4% (1347 of 30 990 births) during the second half of 2012 to 1.8% (529 of 29 605 births) during the first half of 2018; the proportion more than 34 years old rose from 21.1% (6533 births) to 25.5% (7552 births). The proportion of women with diabetes increased from 6.4% (1988 births) to 13.1% (3885 births) (Box 1). During the same period, the proportion of iatrogenic births beyond 37 weeks’ gestation increased from 40.0% to 49.6% of all births; the proportion of spontaneous vaginal births beyond 37 weeks’ gestation declined from 44.0% to 36.1% (Box 2).

Box 1 **Characteristics of women who gave birth in Queensland, July 2012 – June 2018**

**Table:** 

	Birth year							
Characteristics	July–Dec 2012	2013	2014	2015	2016	2017	Jan–June 2018	Relative change*
All births	30 990	62 387	62 024	60 739	60 902	58 406	29 605	–
Age (years)								
Under 20	1347 (4.4%)	2226 (3.6%)	1944 (3.1%)	1706 (2.8%)	1439 (2.4%)	1363 (2.3%)	529 (1.8%)	–59%
20–34	23 110 (74.6%)	46 463 (74.5%)	46 530 (75%)	45 473 (74.9%)	45 610 (74.9%)	43 143 (73.9%)	21 524 (72.7%)	–3%
Over 34	6533 (21.1%)	13 698 (22.0%)	13 550 (21.9%)	13 560 (22.3%)	13 853 (22.8%)	13 900 (23.8%)	7552 (25.5%)	+21%
Body mass index								
Underweight (< 18 kg/m^2^)	2110 (6.8%)	4346 (7.0%)	4322 (7%)	4495 (7.4%)	4268 (7.0%)	3807 (6.5%)	1854 (6.3%)	–8%
Healthy (18–24.9 kg/m^2^)	15 896 (51.3%)	32 356 (51.9%)	31 999 (51.6%)	31 203 (51.4%)	31 188 (51.2%)	29 465 (50.5%)	14 566 (49.2%)	–4%
Overweight (25–29.9 kg/m^2^)	7136 (23.0%)	14 016 (22.5%)	13 915 (22.4%)	13 553 (22.3%)	13 703 (22.5%)	13 260 (22.7%)	6995 (23.6%)	+3%
Obese (≥ 30.0 kg/m^2^)	5848 (18.9%)	11 669 (18.7%)	11 788 (19%)	11 488 (18.9%)	11 743 (19.3%)	11 874 (20.3%)	6190 (20.9%)	+11%
Indigenous Australians	1707 (5.5%)	3485 (5.6%)	3587 (5.8%)	3610 (5.9%)	3580 (5.9%)	3467 (5.9%)	1760 (5.9%)	+8%
Born outside Australia	23 339 (75.3%)	46 865 (75.1%)	45 985 (74.1%)	44 775 (73.7%)	44 053 (72.3%)	41 639 (71.3%)	21 098 (71.3%)	–5%
Smoking (before 20 weeks’ pregnancy)	4265 (13.8%)	8460 (13.6%)	7738 (12.5%)	7122 (11.8%)	6713 (11.0%)	6284 (10.8%)	3063 (10.4%)	–25%
Nulliparous	9360 (30.2%)	19 247 (30.9%)	18 887 (30.5%)	18 305 (30.1%)	18 637 (30.6%)	17 836 (30.5%)	9117 (30.8%)	+2%
Multiple pregnancy	1040 (3.4%)	1924 (3.1%)	1918 (3.1%)	1832 (3.0%)	1729 (2.8%)	1752 (3.0%)	813 (2.8%)	–18%
Diabetes	1988 (6.4%)	4707 (7.5%)	5298 (8.5%)	6328 (10.4%)	6940 (11.4%)	7292 (12.5%)	3885 (13.1%)	+105%
Hypertension	845 (2.7%)	1587 (2.5%)	1754 (2.8%)	1814 (3.0%)	2012 (3.3%)	1950 (3.3%)	968 (3.3%)	+20%
Pre‐eclampsia	642 (2.1%)	1376 (2.2%)	1401 (2.3%)	1237 (2.0%)	1338 (2.2%)	1507 (2.6%)	712 (2.4%)	+16%

* Proportions for January–June 2018 *v* July–December 2012.

Box 2 **Characteristics of births in Queensland, July 2012 – June 2018**

**Table:** 

	Birth year								
Characteristic	July–Dec 2012	2013	2014	2015	2016	2017	Jan–June 2018	Relative change*	*P* ^†^
All births	30 990	62 387	62 024	60 739	60 902	58 406	29 605		
Birth in private hospital	11 578 (38.0%)	23 133 (37.8%)	22 138 (36.4%)	22 019 (36.9%)	21 341 (35.8%)	20 088 (35.1%)	9693 (33.4%)	–12%	< 0.001
Iatrogenic birth, < 34 weeks	432 (1.4%)	830 (1.3%)	853 (1.4%)	782 (1.3%)	796 (1.3%)	797 (1.4%)	401 (1.4%)	0%	0.03
Spontaneous birth, < 34 weeks	460 (1.5%)	850 (1.4%)	819 (1.3%)	806 (1.3%)	783 (1.3%)	699 (1.2%)	348 (1.2%)	–20%	< 0.001
Iatrogenic birth, 34–37 weeks	1062 (3.4%)	1961 (3.1%)	2052 (3.3%)	2083 (3.4%)	2089 (3.4%)	2188 (3.8%)	1061 (3.6%)	+6%	< 0.001
Spontaneous birth, 34–37 weeks	945 (3.1%)	1848 (3.0%)	1718 (2.8%)	1728 (2.8%)	1763 (2.9%)	1614 (2.8%)	791 (2.7%)	–13%	< 0.001
Iatrogenic birth, > 37 weeks	12 403 (40.0%)	25 705 (41.2%)	25 938 (41.9%)	26 587 (43.8%)	27 547 (45.3%)	28 198 (48.3%)	14 678 (49.6%)	+24%	< 0.001
Planned caesarean delivery (no labour), > 37 weeks	5637 (18.2%)	11 717 (18.8%)	11 513 (18.6%)	11 317 (18.7%)	11 609 (19.1%)	11 246 (19.3%)	5835 (19.7%)	+8%	< 0.001
Induced labour, > 37 weeks	6766 (21.8%)	13 988 (22.4%)	14 425 (23.3%)	15 270 (25.1%)	15 938 (26.2%)	16 952 (29%)	8843 (29.9%)	+37%	< 0.001
Spontaneous birth, > 37 weeks	15 688 (50.6%)	31 193 (50%)	30 644 (49.3%)	28 753 (47.4%)	27 924 (45.8%)	24 910 (42.5%)	12 326 (41.5%)	–18%	< 0.001
Emergency caesarean delivery, > 37 weeks									
Fetal distress, > 37 weeks	994 (3.2%)	2004 (3.2%)	2051 (3.3%)	2095 (3.5%)	1939 (3.2%)	2109 (3.6%)	1127 (3.8%)	+19%	< 0.001
Non‐progressive labour, > 37 weeks	1303 (4.2%)	2493 (4.0%)	2277 (3.7%)	2189 (3.6%)	2159 (3.6%)	2068 (3.5%)	1028 (3.5%)	–17%	< 0.001
Other reasons, > 37 weeks	6748 (21.8%)	13 981 (22.4%)	13 792 (22.2%)	13 660 (22.5%)	13 985 (23.0%)	13 462 (23.1%)	7000 (23.6%)	+8%	< 0.001
Spontaneous vaginal birth, > 37 weeks	13 628 (44.0%)	27 115 (43.5%)	26 659 (43.0%)	24 997 (41.2%)	24 309 (39.9%)	21 665 (37.1%)	10 682 (36.1%)	–18%	< 0.001

* Proportions for January–June 2018 *v* July–December 2012.

† Cochrane–Armitage tests.

### Birth outcomes

The proportion of births that did not satisfy our criteria for a positive birth outcome increased from 27.1% (8404 births) during the second half of 2012 to 30.5% (9041 births) during the first half of 2018. The proportion of infant deaths declined from 0.9% (285 births) to 0.7% (219 births); the proportion for which birth trauma was recorded increased from 0.8% (240 births) to 1.4% (399 births). The proportion of births for which maternal haemorrhage was recorded increased from 6.8% (2097 births) in the second half of 2012 to 10.9% (3223 births) during the first half of 2018 (Box 3).

Box 3 **Birth outcomes for births in Queensland, July 2012 – June 2018**

**Table:** 

	Birth year								
Characteristic	July–Dec 2012	2013	2014	2015	2016	2017	Jan–June 2018	Relative change*	*P* ^†^
All births	30 990	62 387	62 024	60 739	60 902	58 406	29 605		
Composite birth outcome									
Positive	22 586 (72.9%)	45 544 (73.0%)	45 140 (72.8%)	43 930 (72.3%)	43 169 (70.9%)	40 774 (69.8%)	20 564 (69.5%)	–6%	< 0.001
Non‐positive	8404 (27.1%)	16 843 (27.0%)	16 884 (27.2%)	16 809 (27.7%)	17 733 (29.1%)	17 632 (30.2%)	9041 (30.5%)	+13%	< 0.001
Infant death (stillbirth or neonatal death)	285 (0.9%)	556 (0.9%)	519 (0.8%)	490 (0.8%)	448 (0.7%)	443 (0.8%)	219 (0.7%)	–20%	< 0.001
Admission to special care nursery/neonatal intensive care unit	5600 (18.1%)	11 119 (17.8%)	10 804 (17.4%)	10 804 (17.4%)	11 077 (18.2%)	11 173 (19.1%)	5614 (19.0%)	+5%	< 0.001
Apgar score < 7	693 (2.2%)	1451 (2.3%)	1559 (2.5%)	1512 (2.5%)	1541 (2.5%)	1476 (2.5%)	712 (2.4%)	+7%	< 0.001
Hypoxic ischaemic encephalopathy	10 (< 0.1%)	12 (< 0.1%)	14 (< 0.1%)	22 (< 0.1%)	26 (< 0.1%)	29 (0.1%)	9 (< 0.1%)	0	0.01
Birth trauma	240 (0.8%)	450 (0.7%)	473 (0.8%)	515 (0.9%)	663 (1.1%)	743 (1.3%)	399 (1.4%)	+75%	< 0.001
Hypoxia	68 (0.2%)	146 (0.2%)	122 (0.2%)	103 (0.2%)	98 (0.2%)	61 (0.1%)	51 (0.2%)	0	< 0.001
Other conditions	2083 (6.7%)	4368 (7%)	4299 (6.9%)	3805 (6.3%)	4034 (6.6%)	3909 (6.7%)	2094 (7.1%)	+5%	0.17
Perineum damage	480 (1.6%)	975 (1.6%)	1092 (1.8%)	1036 (1.7%)	1031 (1.7%)	945 (1.6%)	421 (1.4%)	–8%	0.28
Maternal haemorrhage	2097 (6.8%)	4359 (7.0%)	4730 (7.6%)	5078 (8.4%)	5676 (9.3%)	5757 (9.9%)	3223 (10.9%)	+61%	< 0.001
Rupture of uterus	9 (< 0.1%)	17 (< 0.1%)	19 (< 0.1%)	18 (< 0.1%)	18 (< 0.1%)	19 (< 0.1%)	16 (0.1%)	+87%	0.07
Retained placenta	150 (0.5%)	319 (0.5%)	322 (0.5%)	274 (0.5%)	265 (0.4%)	242 (0.4%)	96 (0.3%)	–33%	< 0.001

* Proportions for January–June 2018 *v* July–December 2012.

† Cochrane–Armitage tests.

### Birth costs

The mean cost per birth (all funders) increased from $20 471 (standard deviation [SD], $17 513) during the second half of 2012 to $30 000 (SD, $22 323) during the first half of 2018 (Box 4). The mean cost to public hospital funders increased from $14 359 (SD, $18 857) to $24 638 (SD, $24 638); to the PBS from $48 (SD, $307) to $139 (SD, $1478); and to private health insurers from $2798 (SD, $5091) to $3464 (SD, $6776) (Box 5).

Box 4 **The costs and value of births in Queensland, July 2012 – June 2018**

**Table:** 

	Birth year							
Characteristic	July–Dec 2012	2013	2014	2015	2016	2017	Jan–June 2018	Relative change*
Cost per birth, mean (SD)*	$20 471 (17 513)	$23 135 (24 695)	$25 640 (22 752)	$25 843 (26 143)	$26 383 (24 137)	$28 736 (24 363)	$30 000 (22 323)	+47%
Positive birth outcome*	$16 909 (6 733)	$18 967 (8 345)	$21 708 (8 746)	$22 199 (7 969)	$22 479 (8 935)	$24 515 (9 481)	$25 755 (11 157)	+52%
Non‐positive birth outcome*	$29 782 (29 495)	$34 056 (43 133)	$35 896 (39 021)	$35 142 (46 311)	$35 689 (40 720)	$38 269 (39 998)	$39 403 (34 615)	+32%
Total costs: all births (A)	$1 306 751 392^†^	$1 491 362 130	$1 639 649 869	$1 621 218 253	$1 660 858 232	$1 737 503 654	$1 840 845 946^†^	+41%
Positive birth outcomes (B)	45 172^†^	45 544	45 140	43 930	43 169	40 774	41 092^†^	–9%
Cost per positive outcome (A/B)	$28 928	$32 746	$36 324	$36 905	$38 473	$42 613	$44 798	+55%

* Absolute values for January–June 2018 *v* July–December 2012.

† Actual numbers (A, B) doubled to estimate annual costs.

Box 5 Mean costs per birth for births in Queensland, July 2012 – June 2018, by funder*

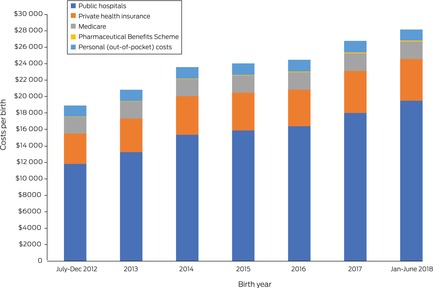

* The data for this graph are included in the Supporting Information, table 1.

The proportion of births for which total costs (all funders) did not exceed $18 000 declined from 65% (20 144 births) during the second half of 2012 to 14% (4145 births) during the first half of 2018; the proportion for which costs were $25 000 or more increased from 14% (4339 births) to 44% (13 026 births) (Box 6).

Box 6 **Distribution of total costs per birth for births in Queensland, July 2012 – June 2018**

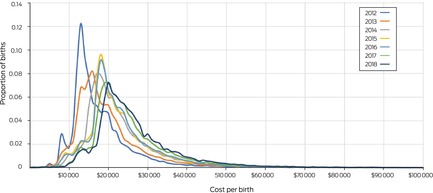

The annual total costs for all pregnancies increased from $1.31 billion for the second half of 2012 to $1.84 billion for the second half of 2018. The cost per positive outcome achieved (value) increased from $28 928 to $44 798, the change was linked with the decline in the number of births with positive outcomes and the increase in total costs of care (Box 4).

In our mixed effects linear analysis, the mean total cost per birth in the second half of 2018 was $9493 higher (99.9% confidence interval [CI], $8930–10 056) than in the first half of 2012. During 2012–18, the estimated differences from the mean costs for spontaneous births beyond 37 weeks’ gestation were $51 398 (99.9% CI, $50 437–52 359) for iatrogenic births before 34 weeks’ gestation, $15 652 (99.9% CI, $14 977–16 327) for iatrogenic births at 34–37 weeks’ gestation, and $2794 (99.9% CI, $2544–3044) for iatrogenic births beyond 37 weeks’ gestation (Box 7).

Box 7 **Total costs per birth for births in Queensland, July 2012 – June 2018, adjusted for time and demographic and characteristics of the women: mixed effects linear model**

**Table:** 

Variable	Estimate	99.9% confidence interval
Intercept	$17 651	$16 789–18 513
Year		
July–Dec 2012	Reference	
2013	$2876	$2396–3356
2014	$5423	$4943–5903
2015	$5507	$5023–5991
2016	$6001	$5517–6485
2017	$8109	$7622–8596
Jan–June 2018	$9493	$8930–10 056
Mother's age (years)		
Under 20	$1698	$1013–2383
20–34	Reference	
Over 34	–$49	–$332 to $234
Body mass index		
Underweight (< 18 kg/m^2^)	$1206	$745–1667
Healthy (18–24.9 kg/m^2^)	Reference	
Overweight (25–29.9 kg/m^2^)	$757	$467–1047
Obese (≥ 30.0 kg/m^2^)	$2012	$1699–2325
Country of birth (overseas)	$1257	$994–1520
First pregnancy	$478	$221–735
Singleton pregnancy	–$3794	–$4518 to –$3070
Diabetes	$1614	$1219–2009
Hypertension	$1928	$1250–2606
Pre‐eclampsia	$7211	$6438–7984
Birth type/gestation		
Spontaneous > 37 weeks	Reference	
Iatrogenic < 34 weeks	$51 398	$50 437–52 359
Spontaneous < 34 weeks	$56 720	$55 782–57 658
Iatrogenic 34–37 weeks	$15 652	$14 977–16 327
Spontaneous 34–37 weeks	$10 186	$9498–10 874
Iatrogenic > 37 weeks	$2794	$2544–3044
Congenital anomalies	$6372	$5928–6816

## Discussion

We found that the mean cost per birth for maternity care in Queensland increased during 2012–18, after adjusting for clinical and demographic characteristics, by $9493. The total costs for all births increased from $1.31 to $1.84 billion, despite a slight decline in the annual number of births. The proportion of births with non‐positive outcomes rose from 27.1% in the second half of 2012 to 30.5% in the first half of 2018. Specifically, the proportions of births for which maternal haemorrhage or birth trauma were recorded increased during 2012–18, and the proportion of spontaneous vaginal births beyond 37 weeks declined from 44% to 36%.

Several authors have advocated promoting better value in maternity care, and some have proposed specific strategies, including better use of contraception, tailoring perinatal care to the needs of individual women, value‐based payments rather than fixed fees for services, and greater use of birth centres.^10^, ^11^ These suggestions, however, have largely been based upon opinion, rather than based on evidence about how value changed over time and why. Our study is the first to quantify change in the value of care.

Evidence for effective and cost‐effective treatment or prevention options is available for many of the contributors to increasing costs we identified. For example, caseload midwifery can be cost‐effective and reduce the likelihood of an iatrogenic birth compared with standard midwifery care.^19^, ^20^ The federally funded Australian Preterm Prevention Alliance is seeking to reduce the rate of pre‐term births by 20%.^21^ Healthy weight and excess gestational weight gain are general population health problems,^22^ and evidence for the effectiveness and cost‐effectiveness of healthy lifestyle interventions during pregnancy have been reported in Australia and overseas.^23^, ^24^ Level I evidence from 117 randomised trials of specific lifestyle interventions indicates that diet interventions reduce the rates of gestational diabetes mellitus (odds ratio [OR] for outcome, 0.61; 95% CI, 0.45–0.82), pre‐term delivery (OR, 0.43; 95% CI, 0.22–0.84), neonatal intensive care admission (OR, 0.68; 95% CI, 0.48–0.95), any adverse maternal outcome (OR, 0.75; 95% CI, 0.61–0.92), and any neonatal outcome (OR, 0.44; 95% CI, 0.26–0.72).^23^ Physical activity interventions reduce pregnancy complication rates and that of the need for caesarean delivery (OR, 0.85; 95% CI, 0.75–0.95).^23^ Antenatal lifestyle interventions are recommended by the United States Prevention Task Force^25^ and in a 2014 case for action proposal submitted to the National Health and Medical Research Council;^26^ early identification of pre‐eclampsia can also be cost‐effective.^27^, ^28^ Despite the many challenges for pre‐conception care, evidence that health service improvements can reduce costs and improve health care outcomes has been reported.^29^

Coordinated efforts by clinicians, health services, and public health officials will be needed to improve value care, as is a national approach to reducing documented variations in costs between Australian hospitals.^30^ The rising caesarean delivery rate overseas^4^, ^14^ and its long term implications for women and their children^31^ has been described; many Australian women report dissatisfaction with several aspects of maternity care;^32^ and in this article we report that maternity care costs are increasing. As such growth is not sustainable, given caps on federal hospital funding and generally tightening post‐pandemic economic policy, all involved must work together to implement effective prevention and treatment strategies for providing consistently high value care.

### Limitations

Our analysis of population‐based administrative data comprehensively captured maternity care costs, rather than narrowly focusing on single episodes of care. However, the primary outcome assessed was not based on outcomes reported by pregnant women, which would be ideal for evaluating value. Outcomes reported by patients are incorporated into service assessments in some countries;^33^ National Health Service England, for example, requires that patient‐reported outcomes be assessed before and after hip and knee replacement surgery undertaken in its services.^34^ Our findings may not be entirely generalisable to the rest of Australia, but intervention rates during pregnancy in Queensland are similar to those in other states.^35^

### Conclusion

We found that the costs of maternity care have increased in Queensland, and that many adverse birth outcomes have become more frequent. Continuing cost increases of the magnitude we report are unsustainable. Broad clinical collaboration, effective prevention and treatment strategies, as well as maternal health services focused on all dimensions of value (patient and provider experience, quality of care and outcomes, efficiency, and sustainability), are needed to ensure the quality and viability of maternity care.

## Open access

Open access publishing facilitated by University of Technology Sydney, as part of the Wiley ‐ University of Technology Sydney agreement via the Council of Australian University Librarians.

## Competing interests

No relevant disclosures.

## Supporting information


Supplementary methods and results
