# Supplementary material for: The value of maternity care in Queensland, 2012–18, based on an analysis of administrative data: a retrospective observational study
Source: Med J Aust. 2023 Nov 8;219(11):535–41. doi: 10.5694/mja2.52156 (PMC10952409; doi:10.5694/mja2.52156)
Supplement: Supplementary file 1 — Supplementary methods and results [file MJA2-219-535-s001.pdf]

MJA
The Medical Journal of Australia

Supporting Information

Supplementary methods and results

This appendix was part of the submitted manuscript and has been peer reviewed. It is posted as supplied by the authors.

Appendix to: Callander EJ, Enticott JC, Eklom B, et al. The value of maternity care in Queensland, 2012–18, based on an analysis of administrative data: a retrospective observational study. *Med J Aust* 2023; doi: 10.5694/mja2.52156.

Structure of maternity health care in Australia

Australia has a complex, multi-payer system that aims to provide universal health care. Health care is provided through community-based, privately provided, but federal government-funded primary and secondary care providers; this system is referred to as 'Medicare'. The federal government also subsidises pharmaceutical costs through the 'Pharmaceutical Benefits Scheme' (PBS). Out-of-pocket fee for patients can remain for all services and treatments subsidized by Medicare and the PBS. State governments provide public hospital care, including acute inpatient, emergency department, and outpatient care. Public hospitals are jointly funded by the state and federal governments, and no out-of-pocket fees are payable for public hospital services. Private hospital care is provided by private providers, partially funded by private health insurers for people who buy private health insurance.

Maternity care in Australia is provided in public and private hospitals. In the private system, a woman chooses a private obstetrician for antenatal care and to attend or oversee her birth at a public or (usually) private or hospital. In the public system, care provided by public hospital midwives or obstetricians includes antenatal, intrapartum, and postnatal care up to six weeks after giving birth. In both models of care, an initial consultation with a general practitioner is also required, and diagnostic, screening, and pathology tests may also be required in primary care.

Care in public hospital inpatient and outpatient departments is funded by public hospital funding agreements; care in the community, including private obstetrician consultations, and all procedures and services provided in private hospitals are co-funded by Medicare and out-of-pocket fees. Private health insurers fund the cost of private hospital stays and may partly reimburse out-of-pocket fees for services provided in private hospitals. Some examples of providers and funders of care for women seeking public or private hospital care in Australia are outlined in table 1.

Table 1. Providers of services during the perinatal time period in Australia, and sources of funding

**Service**	**Public Care**		**Private care**	
	Provider	*Funder*	*Provider*	*Funder*
Antenatal check-ups	Public hospital midwife	Public Hospital Funders	Private obstetrician	Medicare, out-of-pocket fees
Blood serology tests	Community-based pathology service	Medicare, out-of-pocket fees	Community-based pathology service	Medicare, out-of-pocket fees
Ultrasound	Community-based radiology service	Medicare, out-of-pocket fees	Community-based radiology service	Medicare, out-of-pocket fees
Intrapartum care	Public hospital midwife and obstetrician	Public Hospital Funders	Private obstetrician	Medicare, out-of-pocket fees (private health insurance may reimburse for out-of-pocket fees)
Postpartum in-hospital care	Public hospital midwife	Public Hospital Funders	Private hospital	Private health insurance
Neonatal Intensive Care	Public hospitals	Public Hospital Funders	Public hospitals	Public Hospital Funders

Table 2. Cost per birth, Queensland, 2012–18

A. Mean (standard deviation)

Funding source	Birth year July-Dec 2012	2013	2014	2015	2016	2017	Jan-June 2018
Public hospital	$14,359 ($15,195)	$16,634 ($15,766)	$18,683 ($16,787	$19,078 ($19,875)	$19,718 ($19,261)	$21,657 ($21,123	$23,125 ($20,416)
Private health insurance	$2798 ($5806)	$3096 ($6141)	$3525 ($7392)	$3321 ($7774)	$3171 ($7501)	$3,572 ($7533)	$3464 ($7991)
Medicare	$2021 ($1864)	$2067 ($1892)	$2091 ($1857)	$2080 ($1815)	$2109 ($1814)	$2,137 ($1817)	$2107 ($1766)
Pharmaceutical Benefits Scheme	$48 ($307)	$54 ($443)	$58 ($434)	$64 ($488)	$102 ($652)	$122 ($779)	$139 ($1478)
Out-of-pocket costs	$1305 ($1961)	$1352 ($2028)	$1351 ($2053)	$1373 ($2103)	$1396 ($2147)	$1376 ($2141)	$1312 ($2087)
**TOTAL**	$20,471 ($17,513)	$23,135 ($24,695)	$25,640 ($22,752)	$25,843 ($26,143)	$26,383 ($24,137)	$28,736 ($24,363)	$30,000 ($22,323)

B. Median (interquartile range)

Funding source	Birth year July-Dec 2012	2013	2014	2015	2016	2017	Jan-June 2018
Public hospital	$12,030 ($528–18,479)	$14,542 ($610–20,169)	$17,088 ($1,940–22,873)	$17,549 ($3,180–23,385)	$17,778 ($4,287–23,733)	$19,556 ($8,676–26,237)	$21,097 ($16,038 – $28,120)
Private health insurance	$0 ($0–6,630)	$0 ($0–6,822)	$0 ($0–8,387)	$0 ($0–7,317)	$0 ($0–6,932)	$0 ($0–6,905)	$0 ($0–6,185)
Medicare	$1325 ($830–2,843)	$1403 ($882–2,817)	$1457 ($936–2,798)	$1479 ($955–2,738)	$1504 ($986–2,761)	$1531 ($1,014–2,771)	$1531 ($1,024–2,694)
Pharmaceutical Benefits Scheme	$0 ($0–7)	$0 ($0–7)	$0 (0–7)	$0 ($0–10)	$0 ($0–17)	$0 ($0–25)	$0 ($0–29)
Out-of-pocket costs	$197 ($31–2,713)	$219 ($43–2,817)	$226 ($50–2,694)	$228 ($50–2,675)	$241 ($60–2,641)	$249 ($66–2,279)	$263 ($75–2,478)
Total	$16,326 ($13,205–21,218)	$18,511 ($14,946–23,649)	$21,088 ($17,399–26,608)	$21,373 ($18,081–26,815)	$21,758 ($18,267–27,284)	$23,792 ($19,714–29,886)	$25,058 ($20,650–31,577)
